# Comparison of pathogen detection performance between metagenomic next-generation sequencing and conventional culture in organ preservation fluids and recipient wound drainage fluids

**DOI:** 10.3389/fcimb.2025.1563962

**Published:** 2025-08-11

**Authors:** Jiyuan Li, Wenjia Yuan, Chen Gao, Lei Liu, Lei Song, Wei Cao, Xuejing Zhu, Yachun Han, Ruobing Liang, Gongbin Lan, Shaojie Yu, Yu Wang, Liang Tan, Helong Dai, Xubiao Xie, Longkai Peng, Fenghua Peng

**Affiliations:** ^1^ Department of Kidney Transplantation, Center of Organ Transplantation, The Second Xiangya Hospital of Central South University, Changsha, Hunan, China; ^2^ Department of Medical Laboratory, The Second Xiangya Hospital of Central South University, Changsha, Hunan, China; ^3^ Department of Nephrology, The Second Xiangya Hospital of Central South University, Changsha, Hunan, China; ^4^ Department of Scientific Affairs, Hugobiotech Co., Ltd., Beijing, China

**Keywords:** kidney transplantation, metagenomic next-generation sequencing, preservation fluid, drainage fluid, donor-derived infection, microbial culture

## Abstract

**Background:**

Prompt identification and management of donor-derived infections post-kidney transplantation are critical. This study aims to assess the effectiveness of metagenomic next-generation sequencing (mNGS) in detecting pathogens within donor organ preservation fluids and recipient wound drainage fluids, with a comparison made against conventional culture methods.

**Methods:**

This study involved 141 kidney transplant patients (May 1st, 2020 to Jan 31st, 2024). Donor organ preservation fluids and recipient wound drainage fluids were collected and analyzed by mNGS and conventional culture. Pathogen detection differences between mNGS and culture were evaluated. The antibiotic adjustment and infectious complications of the recipients were recorded.

**Results:**

For organ preservation fluids, the positive rate of convention culture were lower than that of mNGS (24.8% (35/141) vs 47.5% (67/141), p<0.05). For recipient wound drainage fluids, the positivity rate of convention culture were lower than that of mNGS (2.1% (3/141) vs 27.0% (38/141), p<0.05). Compared to traditional culture-based methods, mNGS demonstrated a significantly higher positive detection rate for the combination of ESKAPE pathogens and/or fungi (28.4% (40/141) vs 16.3% (23/141) *p*< 0.05). Of the pathogens detected through convention culture, mNGS was capable of detecting 79.2% (19/24) of combinations comprising Enterobacteriaceae and non-fermenting bacteria, yet it detected only 22.2% (2/9) of Gram-positive bacteria, and 55.6% (5/9) of fungi. Certain clinically atypical pathogens, mainly *Mycobacterium*, *Clostridium tetanus*, and parasites, can solely be detected via mNGS. The rehospitalization rate due to infections was 13.5% (19/141), while the donor-derived infection rate amounted to 2.8% (4/141). Guided by mNGS and bacterial culture results, adjustments were made to antibiotic administration, with no severe vascular complications arising.

**Conclusions:**

By employing mNGS to analyze drainage fluids and organ preservation fluids, highly pathogenic and atypical pathogenic microorganisms can be rapidly identified with high throughput. While limitations exist in detecting fungi and Gram-positive bacteria, mNGS are need to be jointly applied with conventional culture under current conditions.

## Introduction

Donor-derived pathogens constitute a significant cause of post-kidney transplantation infections, posing challenges to transplant surgeon and nephrologists for an extended period. Although lung infections and urinary tract infections are the most prevalent infections after transplantation, arterial anastomotic rupture and infectious aneurysm are the most alarming complications in the early postoperative stage. These complications often lead to the resection of graft, arterial anastomotic hemorrhage, or recipient death (Wan et al., 2017). Consequently, nephrologists are compelled to perform repeated pathogen cultures and administer broad-spectrum antibiotics prophylactically. However, the lengthy duration and high false-negative rates of conventional bacterial cultures not only impede the prompt administration of sensitive antibiotics but also potentially result in antibiotic misuse (Anesi et al., 2019).

Early and precise identification of pathogens constitutes a crucial aspect of anti-infective treatment following kidney transplantation. Metagenomic next-generation sequencing (mNGS) plays an increasingly important role in the prevention and treatment of various infections (Gu et al., 2019). Currently, the mNGS approach leverages cell-free DNA (cfDNA) isolated from blood (Gu et al., 2021), bronchoalveolar lavage fluid (Chen et al., 2021), and cerebrospinal fluid (Li et al., 2023) to swiftly furnish information on the species of pathogenic microorganisms, enhancing clinical diagnosis and guiding effective therapeutic interventions. Therefore, since 2020, we have integrated the mNGS method with culture techniques after kidney transplantation to evaluate its potential for improving diagnostic accuracy and clinical management. Drainage fluid may contain pathogens from the vascular anastomosis site, providing an early indications of potential vascular infections. Consequently, we conducted comparisons not only between mNGS and conventional culture not only in organ preservation fluids but also in postoperative recipient wound drainage fluids.

## Materials and methods

### Study design and ethics statement

In this retrospective study, we included 141 adult recipients (> 18 years of age) who underwent their first kidney transplantation at the Organ Transplantation Center, Second Xiangya Hospital of Central South University, China, from May 1st, 2020 to Jan 31st, 2024. All patients were followed up for at least 3 months, and relevant data were collected regarding hospitalizations due to infections within the first 3 months post-surgery. The deceased donors (DD), including 133 donation after brain death (DBD) donors, 6 donation after circulatory death (DCD) donors, and 2 donation after brain and circulatory death (DBCD) donors were automatically allocated from The China Organ Transplant Response System (COTRS) according to the national policies. The clinical characteristics were shown in **Table 1**. All recipients who were included in the study provided written informed consent. Clinical data, including conventional microbial tests and antimicrobial management before and after mNGS, were obtained for subsequent statistical analysis. This study has been reviewed and approved by the Medical Research Ethics Committee of the Second Xiangya Hospital of Central South University, with the ethics approval number LYF20240010.

**Table 1:** Demographic characterization.

Characteristic		Donor(n)	Recepient(n)
Sex (n)	Man	107	107
	Woman	34	34
Age (years,n)	<30	33	20
	30-60	88	120
	>60	20	1
	Mean ± SD	42.32±20.38	41.33±12.00
Blood Type(n)	A	42	43
	B	36	37
	O	51	49
	AB	12	12
Weight (kg, Mean ± SD)		60.46±23.65 (n=131)	62.52±13.25 (n=141)
Height (cm, Mean ± SD)		156.36±26.22 (n=129)	164.54±9.16 (n=141)
BMI (Donor,n=129)(Recepient,n=141)	<18.5	15	13
	18.5-23.9	60	72
	>23.9	54	56
Donation type	DBD	133	
	DCD	6	
	DBCD	2	
Cause of death	Trauma	61	
	Stroke	65	
	Other	15	
Etiology	Unknown		69
	Chronic glomerulonephritis		48
	Diabetic nephropathy		7
	Urolithiasis		6
	Polycystic kidney		3
	Nephrotic syndrome		2
	Gouty nephropathy		2
	Hypertensive nephropathy		2
	Acute progressive nephritis		1
	Pulmonary hemorrhagic nephritis syndrome		1
Fever within 3 days	Yes	54	
	No	37	
	Not recorded	50	
Days in ICU (n=135,6 not recorded)	<3	64	
	3-7	45	
	8-14	16	
	>14	10	
	Mean ± SD	5.61±8.97	

### Sample collection

On the organ preparation table, two tubes of organ preservation fluids (hypertonic citrate purine solution S400, YZB/2173-2015, Shanghai, China, 10 mL each) were collected, 10ml sample is sent for conventional culture and the other 10ml is sent for mNGS. On the 1st, 2nd, and 3rd day after surgery, 10ml of the recipient’s drainage fluid from drainage bag was collected for conventional culture. Driven by cost-effectiveness considerations, we perform mNGS on drainage fluid only on the 1st day after surgery.

### Conventional culture and identification method

Samples were directly inoculated into aerobic culture bottles (BD BACTEC Plus Aerobic/F). All blood culture bottles were loaded onto the BD BACTEC FX instrument (Becton Dickinson, Franklin Lakes, NJ, USA). After a positive signal, the blood culture broth was analyzed by Gram stain and cultured onto a blood agar plate (BIOIVT, Zhengzhou, China) and incubated in 5%CO2 at 35 ± 1°C for 18 to 24h (Thermo Fisher Scientific, USA). If Gram staining showed the presence of fungi, the sample was additionally subcultured on SDA agar plate (BIOIVT, Zhengzhou, China) and incubated at 37°C for 48 h. After incubation, colonies were transferred to a 96-spot steel target plate (Bruker Daltonics, Bremen, Germany) for Matrix Assisted Laser Desorption Ionization Time-of-Flight Mass Spectrometry (MALDI-TOF MS) analysis, and microorganisms were directly identified by MALDI-TOF MS (Bolger et al., 2014).

### Metagenomic sequencing and bioinformatic analysis

The preservation fluid and drainage fluid samples were transported to Hugobiotech Co., Ltd. (Beijing, China) for PACEseq mNGS. Human cells in samples were removed by centrifugation. cfDNA was extracted from the supernatant using QIAamp DNA Micro Kit (QIAGEN, Hilden, Germany) according to the instructions. The extracted cfDNA concentrations were measured by Qubit 4.0 (Thermo Fisher Scientific, MA, USA). Then, qualified metagenomics libraries were constructed and sequenced on Nextseq 550 platform (Illumina, San Diego, USA). In parallel with the clinical samples, positive control and negative control (non-template control, NTC) were also set for mNGS detection with the same procedure and bioinformatics analysis.

Raw metagenomic sequencing datasets were trimmed adapter sequences and filtered low-quality reads (< 35bp) by Trimmomatic (v0.39) (Bolger et al., 2014). After quality control, the reads were mapped to human reference genome GRCh38.p13 to remove host reads by bowtie2 (v2.4.2) (Langmead and Salzberg, 2012) based on kneaddata (v0.7.4) (https://huttenhower.sph.harvard.edu/kneaddata). Bowtie2 was further used to align putative non-human reads generated by kneaddata to Homo sapiens YH1 genome of an Asian individual and those human associated sequences with taxid 9606 in NCBI Nucleotide database. All remaining reads were classified through simultaneous alignment to the reference microbial sequences sourced from NCBI nt database, encompassing archaea, bacteria, viruses and fungi. Sequence alignment process was performed by BLASTN (v2.10.1+) using “megablast” option and only those reads exhibiting unique-alignments to microbial taxa were subsequently counted (Altschul et al., 1990). The positive criteria for the mNGS result were set as follows:

1. For the detected bacteria (Mycobacterium excluded), fungi (Cryptococcus excluded), and parasites: a) genome coverage of the unique reads mapped to this microorganism ranked top10 of the same kind of microbes and the microorganism was not detected in the NTC; or b) RPMsample/RPMNTC was > 10 (RPMNTC≠0).
2. For Mycobacterium tuberculosis, and Cryptococcus: a) the unique reads of this microbe were not detected in NTC but at least 1 specific read was mapped to species; or b) RPMsample/RPMNTC was > 5 (RPMNTC≠0).

### Immunosuppressive therapy and antimicrobial measures

Routine induction immunosuppression was administered to all 141 recipients, consisting of methylprednisolone (500 mg on the day of surgery, then 250 mg daily on postoperative days 1 and 2, followed by 125 mg daily on postoperative days 3 and 4) and oral methylprednisolone (starting at 48 mg from postoperative day 5, tapering to 12 mg/day and maintaining 8 mg every day). Triple immunosuppressive regimen (tacrolimus -mycophenolate mofetil (MMF) -prednisone) was initiated within 24 hours after transplantation and tacrolimus trough concentration maintained at 8–12 ng/ul for the first 3 months after operation.

These transplant recipients received empirical perioperative antibacterial (cefoperazone-sulbactam) and antifungal (caspofungin) prophylaxis, if the microbial cultures of donors before operation were negative. The regimen was typically administered for a duration of 6–8 days until negative results of drainage fluids collected on pod 3 were confirmed. The antimicrobial regimen was further adjusted based on mNGS or bacterial culture and susceptibility testing and consulted with clinical pharmacist. – Conventional application of ganciclovir and bactrim for at least three months to prevent cytomegalovirus and Pneumocystis infection. Mycobacterium tuberculosis detected by mNGS in this study cohort were not specifically treated, except for switching to moxifloxacin and continuing for one month.

### Statistical analysis

Data analysis was performed with SPSS 26.0 and Prism. Categorical variables were expressed as percentages, and continuous variables were expressed as mean ± standard deviation (SD) if normally distributed. Comparative analysis was conducted by Pearson’s test, Fisher’s exact test where appropriate. P < 0.05 was considered significant.

## Results

### Demographic characterization

Among the donors in this study (mean age: 42.32 ± 20.38; male: female = 1: 0.32), the majority passed away due to stroke (46.1%), followed by trauma (43.3%). There were 141 recipients in this study (mean age: 41.33 ± 12.00; male: female = 1:0.32), with chronic glomerulonephritis (34.0%) being the predominant etiology among the patients (**Table 1**).

### Pathogen detection results in different specimens

In the organ preservation fluid, 12 pathogens (39 strains) were detected through culture, including 10 bacteria (30 strains) and 2 fungi (9 strains) (**Supplementary Tables S1**, **S2**). The most prevalent pathogens were *Klebsiella pneumoniae* (6 strains), *Escherichia coli* (6 strains), and *Candida albicans* (6 strains). On the other hand, mNGS identified 35 pathogens (99 strains), including 31 bacterial species (87 strains) and 4 fungal species (12 strains). The four most common pathogens that aligned with the culture results were *Pseudomonas aeruginosa* (14 strains), *Klebsiella pneumoniae* (14 strains), *Escherichia coli* (8 strains), and *Candida albicans* (8 strains).

Wound drainage fluids were cultured daily for the initial 3 days postoperatively, and were identified positive if any of the cultures yielded a positive result. The findings revealed a total of 3 pathogens in the drainage fluid, including 2 bacteria (both *Enterococcus faecalis*) and 1 fungus (*Candida albicans*). Only the first day’s postoperative drainage fluid was subjected to mNGS analysis, which detected 23 pathogens (52 strains), including 20 bacteria (48 strains) and 3 fungi (4 strains). The most prevalent pathogens were *Pseudomonas aeruginosa* (14 strains), *Klebsiella pneumoniae* (8 strains), *Escherichia coli* (3 strains).

### Comparison of positive rates between culture and mNGS in 141 cases

**Figure 1:**
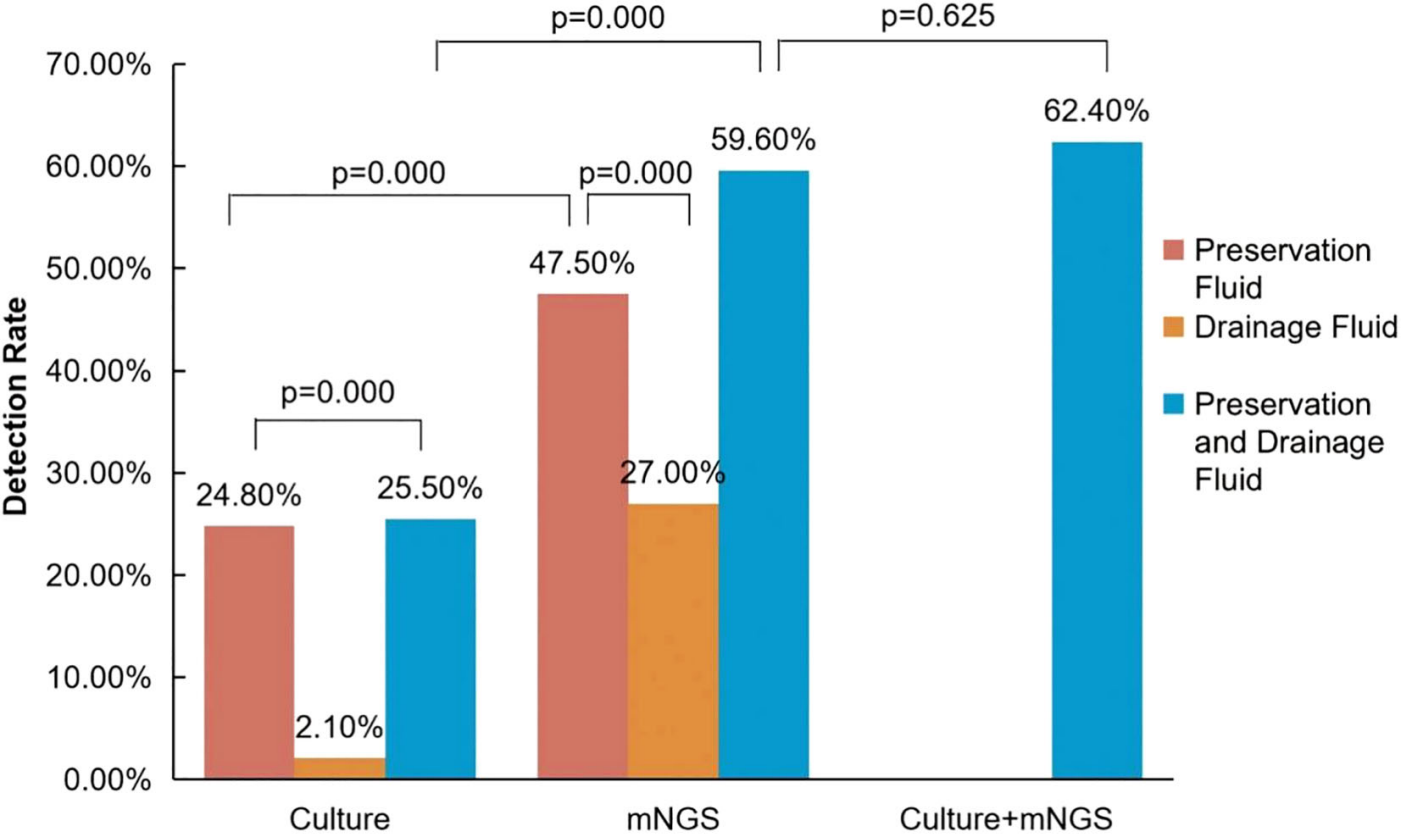
Comparison of positive detection rates between mNGS and conventional culture in organ preservation fluids and wound drainage fluids. The positive rate of pathogens in the preservation solution is represented in brown, while the positive rate in the drainage solution is represented in yellow. When pathogens from the donor preservation solution are counted together with pathogens from the corresponding recipient drainage solution, the positivity rate is indicated in blue. In the culture + mNGS group, a positive result is considered when pathogens are detected either in organ preservation fluid or in recipient wound drainage fluid using either mNGS or conventional culture methods.

Utilization of both mNGS and culture in combination exhibited a higher detection rate compared to culture alone in 141 cases (62.4% (88/141) vs 25.5% (36/141), p<0.05) (**Figure 1**). In addition, mNGS alone demonstrated a higher positive rate compared to culture alone (59.6% (84/141) vs 25.5% (36/141), p<0.05).

When compared to culture, mNGS exhibits a higher positive rate, either in preservation fluid (47.5% (67/141) vs 24.8% (35/141), p<0.05) or in drainage fluid (27.0% (38/141) vs 2.1% (3/141), p<0.05) (**Figure 1**).

### Comparison of the pathogen species between culture and mNGS in different specimens

**Figure 2:**
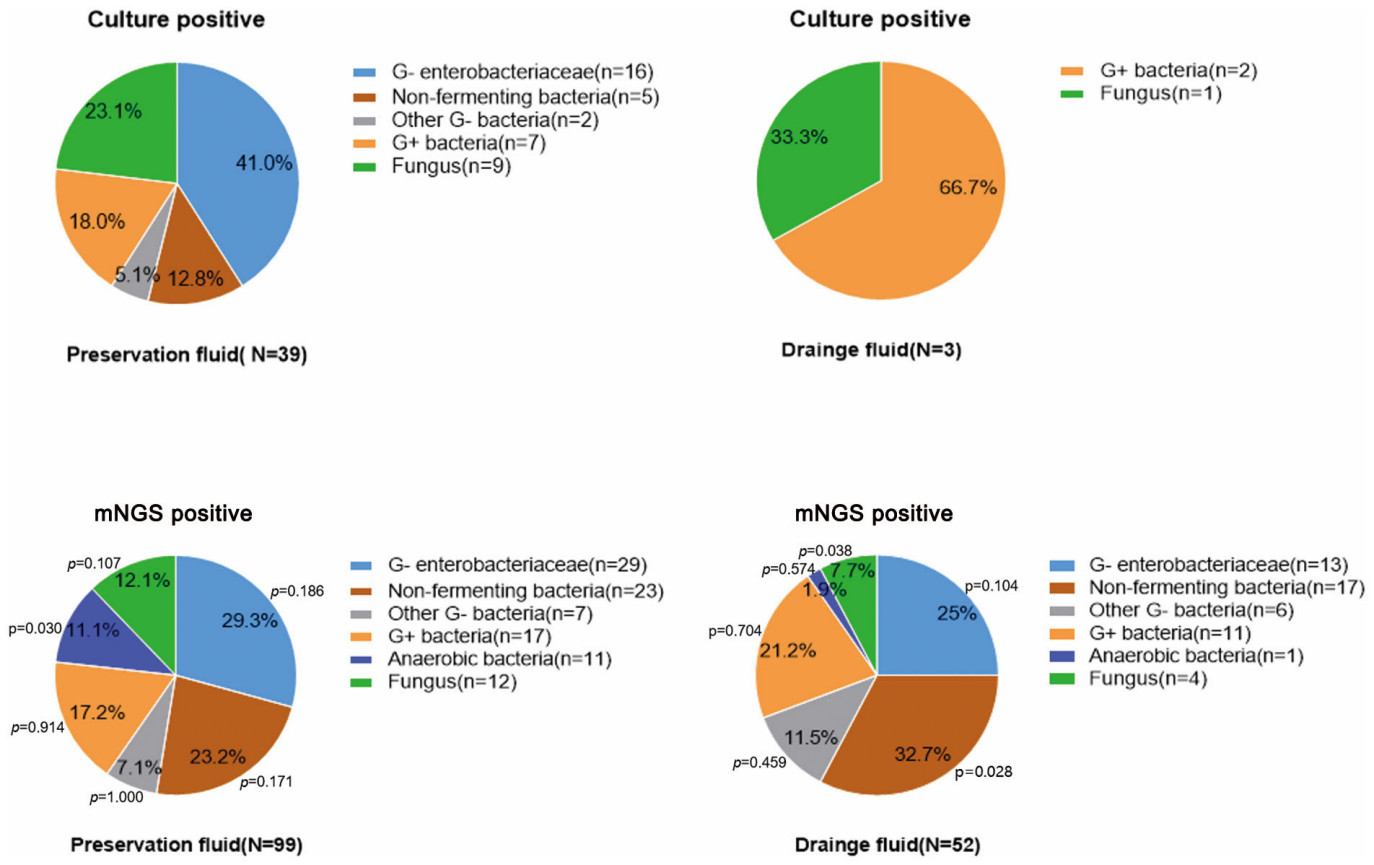
The proportion of pathogens detected by mNGS and conventional culture in organ preservation fluids and wound drainage fluids. Compare the proportion of pathogens detected by culture in the preservation fluids with the proportion of pathogens detected by the mNGS in both the preservation fluids and wound drainage fluids. Since only three cases were positive in the drainage fluid culture, no statistical analysis was conducted.

Among the pathogens detected in the organ preservation fluid, a higher proportion of anaerobic bacteria were identified using mNGS compared to culture (11.1% (11/99) vs 0.0% (0/30), p<0.05). Additionally, compared to the culture in preserved fluid, mNGS in drainage fluid showed a lower proportion of fungi (7.7% (4/52) vs. 23.1% (9/39), p<0.05) (**Table 2**). Enterobacteriaceae and non-fermenting bacteria constitute the major pathogens of Gram-negative bacilli except for the culture results in the drainage fluid. Furthermore, compared with the culture in the preservation fluids, the proportion of non-fermenting bacteria detected in the drainage fluid using the mNGS method was significantly higher (32.7% (17/52) vs 12.8% (5/39), p<0.05) (**Figure 2**; **Supplementary Table S4**).

**Table 2:** Comparison of conventional culture and mNGS in different specimens for detection rate.

Category	Organ Preservation Fluid		Drainage Fluid	
	Culture	mNGS	Culture	mNGS
**Number of patients, n**	141	141	141	141
Positive, n (%)	35 (24.8)	67 (47.5)*	3 (2.1)*	38 (27.0)
ESKAPE-positive, n (%)	14 (14.0)	32 (22.7)*	2 (1.4)*	25 (17.7)
ESKAPE and/or Fungi, n (%)	23 (16.3)	40 (28.4)*	3 (2.1)*	28 (19.9)
**Number of Pathogens Detected, n**	39	99	3	52
Bacteria, n (%)	30 (76.9)	87 (87.9)	2 (66.7)	48 (92.3)*
Fungi, n (%)	9 (23.1)	12 (12.1)	1 (33.3)	4 (7.7)*
Oxygen Requirements				
Aerobes/Facultative, n (%)	30 (76.9)	76 (76.8)	2 (66.7)	47 (90.4)
Anaerobes, n (%)	0 (0.0)	11 (11.1)*	0 (0.0)	1 (1.9)
Gram Stain				
Gram-negative, n (%)	23 (59.0)	59 (59.6)	0 (0.0)	36 (69.2)
Gram-positive, n (%)	7 (17.9)	17 (17.2)	2 (66.7)	11 (21.2)
ESKAPE Pathogens				
Total ESKAPE, n (%)	14 (35.9)	32 (32.3)	2 (66.7)	25 (48.1)
Enterococcus faecium, n	3	0	2	1
Staphylococcus aureus, n	0	1	0	1
Klebsiella pneumoniae, n	6	14	0	8
Acinetobacter baumannii, n	2	3	0	1
Pseudomonas aeruginosa, n	1	14	0	14
Enterobacter cloacae, n	2	0	0	0
ESKAPE and/or Fungi, n (%)	23 (59.0)	40 (40.4)*	3 (100.0)*	28 (53.8)

Compared with the group of culture in organ preservation fluids. Since only three cases were positive in the drainage fluid culture, statistical analysis for some data subsets was not conducted. ^*^p<0.05.

*Enterococcus faecium*, *Staphylococcus aureus*, *Klebsiella pneumoniae*, *Acinetobacter baumannii*, *Pseudomonas aeruginosa*, and *Enterobacter* species (ESKAPE) represents a group of bacteria renowned for their potent pathogenicity and antibiotic-resistance possessing significant clinical importance. In preservation fluid, there was no significant difference in the proportion of ESKAPE whether through culture or mNGS (35.9% (14/39) vs 32.3% (32/99), p>0.05). Fungi, exhibit a high degree of pathogenicity akin to that of ESKAPE pathogens, constituting 23.1% (9/39) of culture-positive cases and 12.1% (12/99) of mNGS-positive cases in preservation fluid, respectively. However, the difference in the two groups was not statistically significant. In the preservation fluid, the proportion of ESKAPE pathogens and/or fungi in the culture group was higher than that in the mNGS group (59.0% (23/39) vs 40.4% (40/99), p<0.05) (**Table 2**).

### Comparison of the ability to detect pathogens between culture and mNGS

We explored this ability by combining the results of the preservation fluid and drainage fluid. It was considered positive if a pathogen was detected in either organ preservation fluid or the corresponding recipient drainage fluid. Among the pathogens detected in culture, mNGS was capable of detecting 79.2% (19/24) of the combinations comprising Enterobacteriaceae and/or non-fermenting bacteria, 22.2% (2/9) of Gram-positive bacteria, and 55.6% (5/9) of fungi.

**Figure 3:**
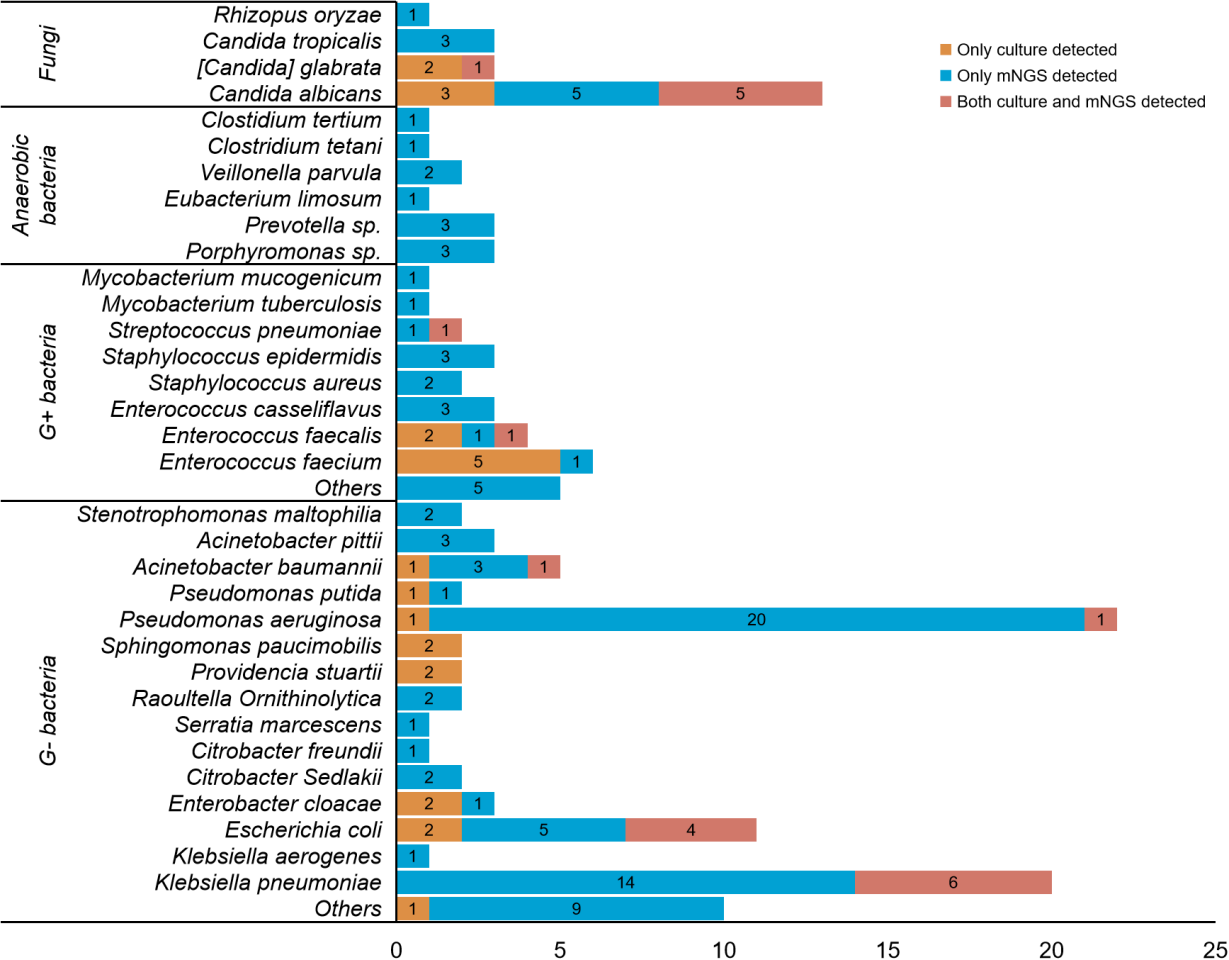
Detailed comparison between culture and mNGS for detecting different pathogens. A total of 145 strains of pathogens were detected in. 141 patients. Only 13.1% (19/145) of the pathogens were detected by both culture and mNGS, with the most common being *Klebsiella pneumoniae* (6 strains, 4.1%), *Candida albicans* (5 strains, 3.4%) and *Escherichia coli* (4 strains, 2.7%) in sequence. 15.9% (23/145) of the pathogens were solely detected in cultures, mainly *Enterococcus faecalis* (5 strains, 3.4%), and *Candida albicans* (3 strains, 2.0%). 71.0% (103/145) of the pathogens were exclusively detected through mNGS, with the top four being *Pseudomonas aeruginosa* (20 strains, 13.6%), *Klebsiella pneumoniae* (14 strains, 9.5%), *Escherichia coli* (5 strains, 3.4%), and *Candida albicans* (5 strains, 3.4%).

**Figure 3**; **Supplementary Table S5** showed the comparison of the performance between culture and mNGS. The results indicated that a total of 145 strains of pathogens were detected, with only 13.1% (19/145) being identified by both culture and mNGS. The most prevalent pathogens detected were *Klebsiella pneumoniae* (6 strains, 4.1%), followed by *Candida albicans* (5 strains, 3.4%) and *Escherichia coli* (4 strains, 2.7%). Furthermore, 15.9% (23/145) of the pathogens were solely detected in cultures, mainly *Enterococcus faecalis* (5 strains, 3.4%), and *Candida albicans* (3 strains, 2.0%). Notably, 71.0% (103/145) of the pathogens were exclusively detected through mNGS, with the top four being *Pseudomonas aeruginosa* (20 strains, 13.6%), *Klebsiella pneumoniae* (14 strains, 9.5%), *Escherichia coli* (5 strains, 3.4%), and *Candida albicans* (5 strains, 3.4%).

Although mNGS generally exhibits greater sensitivity in pathogen detection compared to culture, it still exhibits limitations, especially in the detection of Gram-positive bacteria and fungi. A total of 9 Gram-positive bacteria were cultured, primarily comprising *Enterococcus faecalis* (5 cases) and *Enterococcus faecalis* (3 cases), but only 1 case of *Enterococcus faecalis* was verified by mNGS. Of the remaining 8 cases identified by mNGS, 4 were reported to be other Gram-positive species, 1 was reported to be *Pseudomonas aeruginosa*, 1 was verified to be *Streptococcus pneumoniae*, and 2 did not report any pathogens (**Table 3**). In organ preservation fluids, out of the nine cases of fungi detected by culture, only 5 were confirmed by mNGS.

**Table 3:** Comparison of mNGS detection results of cultured Gram-positive bacteria.

Case	Culture		mNGS	
	Organ Preservation Fluid	Drainage Fluid	Organ Preservation Fluid	Drainage Fluid
1	*Enterococcus faecium*		*Veillonella parvula*	
2	*Enterococcus faecium*		*Veillonella parvula*	*Veillonella parvula*
3	*Enterococcus faecalis*		*Staphylococcus epidermidis*	
4	*Enterococcus faecalis*		*Staphylococcus epidermidis*	
5		*Enterococcus faecium*		
6	*Streptococcus pneumoniae*		*Streptococcus pneumoniae*	*Streptococcus pneumoniae*
7	*Enterococcus faecalis*		*Enterococcus faecalis*	*Enterococcus faecalis*
8	*Enterococcus faecium*			
9		*Enterococcus faecium*	*Pseudomonas aeruginosa*	*Pseudomonas aeruginosa*

### Clinically significant pathogens detected through by mNGS

Some clinically significant pathogens are negative in conventional culture and can only be detected through mNGS, mainly *Mycobacterium*, anaerobic bacteria (such as *Clostridium tetani*), and parasites (such as *Leptospira*), as shown in **Table 4**.

**Table 4:** Clinically significant pathogens only dectected by mNGS.

Pathogens	Organ Preservation Fluid	Drainage Fluid
*Mycobacterium tuberculosis*	2	1
*Mycobacterium mucogenicum*	2	1
*Clostridium tetani*	1	
*Leptospira licerasiae*	1	

### Adjustment of antibiotics and postoperative infection

Under the direction of clinical pharmacists, upon receiving the mNGS results, 15.7% (22/141) of the recipients had their antibiotic treatment regimens altered, with 12.3% (17/141) experiencing an upgrade and 3.5% (5/141) a downgrade. Typically, following consultation with clinical pharmacists, an upgraded treatment regimen involving meropenem, teicoplanin, tigecycline or polymyxin B were prescribed, when Klebsiella pneumoniae Strains were identified by mNGS in 10 cases. Similarly, fluconazole, voriconazole or amphotericin B were prescribed when fungi were identified in 4 cases. Enhanced antibacterial therapy typically spans at least 12 to 14 days, whereas antifungal therapy entails continuous oral administration of triazole antifungal agents for a minimum of 2 months following transplantation.

During the perioperative period, there were no severe complications such as anastomotic rupture or deaths due to infection. Among the 141 recipients, the postoperative infections within 3 months were as follows: 19 readmissions for infections (13.5%, 19/141) (**Supplementary Table S6**), including 8 cases of urinary tract infections (1 combined with pulmonary infection), 4 cases of pulmonary infections (1 combined with urinary tract infection, 1 combined with tuberculosis), 3 case of pulmonary tuberculosis (1 combined with pulmonary infection), 4 cases of fever (2 due to virus infections, 1 due to bacteremia, and 1 of unknown reason), 1 case due to poor wound healing, and 1 case of skin fungal infection. Combining clinical and laboratory examination data, a total of 4 cases were identified as donor-derived infections, including 2 cases of tuberculosis and 2 cases of urinary tract infections (*Escherichia coli* and *Klebsiella pneumoniae, share the same donor*). Based on the results of culture and mNGS, the rate of donor-derived infection in this study was 2.8% (4/141).

## Discussion

Few studies have provided a comprehensive evaluation of the application of mNGS in infections of the perioperative period following renal transplantation. Although culture is the primary approach for detecting infections, it has limitations in detecting uncultivatable or fastidious pathogens (Gu et al., 2021). Therefore, further research is required to improve this embarrassing situation. In this study, we utilized mNGS to identify pathogens in organ preservation fluids. Additionally, we collected and tested wound drainage fluids, which is expected to provide more comprehensive information about donor-derived pathogens.

### mNGS shows a high positive rate

According to our research, mNGS showed a considerably higher positive rate for bacteria and fungi compared to culture. Furthermore, the combination of mNGS with culture further increased the positivity rate to 62.4%, a value that is similar to that of previous studies (Yu et al., 2019). The high detection rate observed may be ascribed to the high sensitivity of mNGS in identifying rare, unexpected, and unknown organisms (Wilson et al., 2019; He et al., 2022). In our research, mNGS detected numerous anaerobic bacteria (such as *Comamonas kerstersii*, *Raoultella ornithinolytica* and *Porphyromonas bennonis*, *Tetanus*) that need appropriate culture media and barely grow in aerobic culture environments, particularly in the cold organ preservation fluids. Additionally, *Mycobacterium* (most typically *Mycobacterium tuberculosis*), as well as some protozoa (such as *Toxoplasma gondii* and *Plasmodium*) require specialized culture media, rendering it challenging to achieve positive results through conventional culture. Conversely, positive results can readily be obtained through mNGS detection. This extraordinary high positivity rate can also partially be attributed to DNAemia, which refers to the detection of bacterial DNA, especially anaerobic bacteria, in the blood of healthy individuals. This suggests that bacteria continuously translocate into the blood but do not always lead to sepsis (Gosiewski et al., 2017). This phenomenon is particularly evident when analyzing drainage fluids, which may contain remnants of decreased microorganisms.

Moreover, in the context of organ transplantation, the overall higher positive rate can also be ascribed to several factors such as unsatisfied aseptic processing environment, adjacent intestine or lung damage during procurement, environmental microbiological contamination in ICUs and non-standard use of antibiotics in the donors (Yu et al., 2019).

### mNGS detection of highly virulent pathogens

ESKAPE pathogens and fungi are highly virulent pathogens and more likely to cause postoperative infections than non-ESKAPE bacteria (Bracale et al., 2009; Berger and Badell, 2017; Tang et al., 2017; Oriol et al., 2018). Our study showed that the positive rate of ESKAPE pathogens by mNGS was significantly higher than that by cultures (28.4% vs 16.3%), indicating that nearly one-third of DD recipients are at high risk of infection.

The pathogens detected in the drainage fluids provide additional insights into infection after initial treatment. Under the premise of prophylactic treatment with broad-spectrum antibiotics and antifungal medicines, the positive rates of ESKAPE and fungi detected through conventional culture in drainage fluids decreased to 2.1%. However, mNGS still demonstrated a notably high positive rate of 19.9%. Among these microbes, *Pseudomonas aeruginosa* and *Klebsiella pneumoniae* were predominant. Given the high virulence of ESKAPE pathogens and fungi, heightened attention to donor-derived contamination and infection is warranted, and intensive treatment of Gram-negative organisms should be continued for an extended period.

### Limitations of mNGS

We observed that mNGS had limitations in detecting several types of pathogens in organ preservation fluids and drainage fluids. For example, the detection rate of fungi by mNGS was lower than that by culture. Only 55.6% (5/9) fungi detected by culture can be confirmed by mNGS. This phenomenon has also been observed by He, P et al, who obtained the similar result, with only 33.3% (4/12) fungi being detected by mNGS (He et al., 2022). Similar limitations were also recorded in the detection of Gram-positive bacteria, as shown in **Figure 3**.

This can be partly attributed to limitations in specimen collection, DNA extraction, polymerase chain reaction (PCR) amplification, mNGS sequencing, and bioinformatics analysis (Boers et al., 2019). At first, cold organ preservation fluids are typically flushed into the procured organ in a large volume, usually totaling 6–7 liters, resulting in significant dilution of microbes within the graft. Trace DNA from pathogens may be overshadowed by a high background of host DNA. Secondly, this could also be partly explained by process of DNA extraction. Some intracellular bacteria, such as *Mycobacterium tuberculosis*, survived more in the host cell and less in body fluids, and some pathogens have a thicker cell wall (such as fungi and Gram-positive bacteria), which can reduce the efficiency of nucleic acid extraction, and the accuracy of identification.

### The clinical application value of mNGS in donor-derived infections

Compared to conventional culture methods, mNGS testing, despite its higher cost, can deliver faster results and greater sensitivity in certain cases. This enables clinicians to adjust treatment plans earlier, potentially leading to significant reductions in overall healthcare costs and improved patient outcomes. However, it is important to note that while mNGS can identify more microorganisms, the issue of false positives in mNGS may lead to overtreatment or unnecessary use of broad-spectrum antibiotics. In clinical practice, mNGS results should be carefully evaluated, and clinical pharmacists should be consulted to focus on adjusting treatment plans for clinically significant pathogens.

Based on the mNGS and culture results, the patients in this study had their medications adjusted timely, with the strength of anti-microbes either increased or decreased. As a result, we achieved good clinical outcomes. There were no cases of serious infectious complications, such as anastomotic rupture or death during the perioperative period. This is a significant contrast to previous reports in China that relied solely on traditional microbiological culture (Wan et al., 2017). Our statistics on readmissions for infections within 3 months after surgery showed an overall infection rate of 13.5%, with a predominance of urinary tract infections. With the treatment guided by culture and mNGS, the confirmed donor-derived infection rate was dramatically reduced to about 2.8%. The main pathogens that can be truly traced back to donor source were tuberculosis and urinary Gram-negative bacillus infections. It is well known that both *Mycobacterium tuberculosis* and upper urinary tract bacterial infections require a long course of treatment. Short-term application of antibiotics during the perioperative period, usually 2 weeks, was insufficient to eliminate such pathogens. Our findings emphasized the importance of mNGS for the detection of *Mycobacterium tuberculosis* and ESKAPE pathogens in preservation fluids and drainage fluids. Additionally, they highlight the need for active intervention in cases of donor-derived urinary tract infections. High infection rate of graft urinary tract within 3 months post-surgery in this study implied the equal importance of prevention from donor-derived urinary tract infections, therefore, further research should be conducted on mNGS and bacterial culture in the urine of recipients.

### Limitations of the study

There are several limitations to this study. First, it is a retrospective study, and due to economic and resource constraints, we collected wound drainage fluid only on the first postoperative day for mNGS testing, potentially resulting in methodological imbalances and sources of bias. Second, our conventional culture methods did not apply appropriate culture media for anaerobic bacteria, Mycobacterium tuberculosis, and specific fungi. The Organ Procurement Organization (OPO) usually excludes donors with active TB based on clinical results, making routine TB cultures not cost-effective. The absence of these specific culture media inevitably introduces bias in the comparison between conventional cultures and mNGS, leading to a reduction in the positive rate by culture. Third, there is currently no clear standard to confirm whether the detected results are truly positive, the presence of potential colonizing bacteria in the specimens may affect the comparison of results, which limits the evaluation of the diagnostic performance of mNGS. At last, this study only involved recipients from a single center in China, which may limit the generalizability of the findings to other healthcare systems.

In conclusion, our study indicates that the application of mNGS in organ preservation fluids and recipient wound drainage fluids aids in the rapid, high-throughput identification of highly virulent and atypical pathogenic microorganisms. With the participation of clinical pharmacists, the outcomes of mNGS facilitate prompt antibiotic adjustments, thereby mitigating severe infection-related vascular complications post-kidney transplantation. Nevertheless, considering the limitations of mNGS in detecting certain highly virulent pathogens, particularly fungi and Gram-positive bacteria in organ preservation fluids and wound drainage fluids, mNGS cannot fully supersede conventional culture and must instead be used in conjunction with them.

## Data Availability

The original contributions presented in the study are included in the article/**Supplementary Material**. Further inquiries can be directed to the corresponding author/s.
