# Supplementary material for: Comparison of pathogen detection performance between metagenomic next-generation sequencing and conventional culture in organ preservation fluids and recipient wound drainage fluids
Source: Front Cell Infect Microbiol. 2025 Aug 11;15:1563962. doi: 10.3389/fcimb.2025.1563962 (PMC12375685; doi:10.3389/fcimb.2025.1563962)
Supplement: Supplementary file 1 [file Presentation1.pptx]

## Slide 1
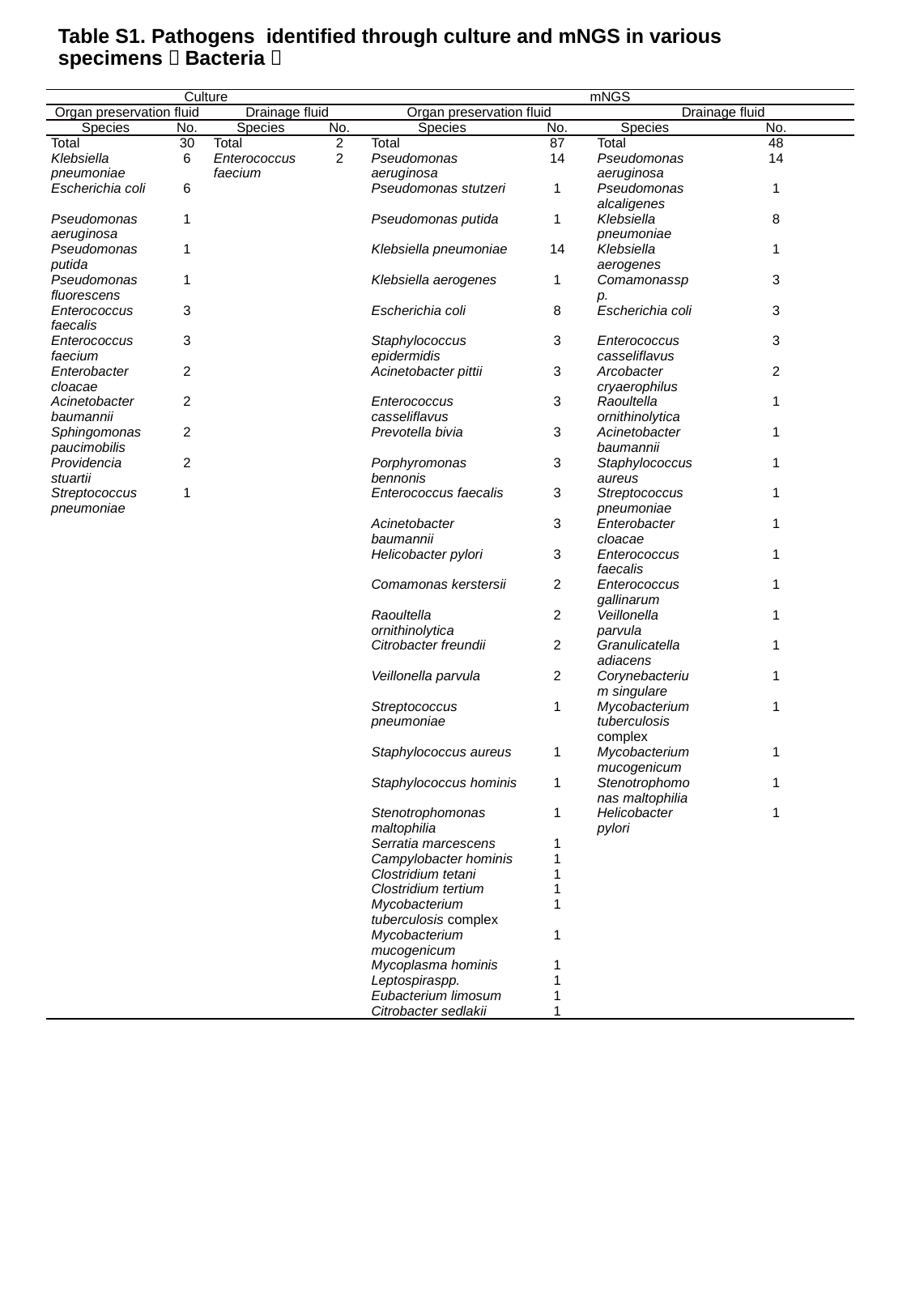

# Table S1. Pathogens identified through culture and mNGS in various specimens（Bacteria）
| Culture | | | | mNGS | | | |
| --- | --- | --- | --- | --- | --- | --- | --- |
| Organ preservation fluid | | Drainage fluid | | Organ preservation fluid | | Drainage fluid | |
| Species | No. | Species | No. | Species | No. | Species | No. |
| Total | 30 | Total | 2 | Total | 87 | Total | 48 |
| Klebsiella pneumoniae | 6 | Enterococcus faecium | 2 | Pseudomonas aeruginosa | 14 | Pseudomonas aeruginosa | 14 |
| Escherichia coli | 6 | | | Pseudomonas stutzeri | 1 | Pseudomonas alcaligenes | 1 |
| Pseudomonas aeruginosa | 1 | | | Pseudomonas putida | 1 | Klebsiella pneumoniae | 8 |
| Pseudomonas putida | 1 | | | Klebsiella pneumoniae | 14 | Klebsiella aerogenes | 1 |
| Pseudomonas fluorescens | 1 | | | Klebsiella aerogenes | 1 | Comamonasspp. | 3 |
| Enterococcus faecalis | 3 | | | Escherichia coli | 8 | Escherichia coli | 3 |
| Enterococcus faecium | 3 | | | Staphylococcus epidermidis | 3 | Enterococcus casseliflavus | 3 |
| Enterobacter cloacae | 2 | | | Acinetobacter pittii | 3 | Arcobacter cryaerophilus | 2 |
| Acinetobacter baumannii | 2 | | | Enterococcus casseliflavus | 3 | Raoultella ornithinolytica | 1 |
| Sphingomonas paucimobilis | 2 | | | Prevotella bivia | 3 | Acinetobacter baumannii | 1 |
| Providencia stuartii | 2 | | | Porphyromonas bennonis | 3 | Staphylococcus aureus | 1 |
| Streptococcus pneumoniae | 1 | | | Enterococcus faecalis | 3 | Streptococcus pneumoniae | 1 |
| | | | | Acinetobacter baumannii | 3 | Enterobacter cloacae | 1 |
| | | | | Helicobacter pylori | 3 | Enterococcus faecalis | 1 |
| | | | | Comamonas kerstersii | 2 | Enterococcus gallinarum | 1 |
| | | | | Raoultella ornithinolytica | 2 | Veillonella parvula | 1 |
| | | | | Citrobacter freundii | 2 | Granulicatella adiacens | 1 |
| | | | | Veillonella parvula | 2 | Corynebacterium singulare | 1 |
| | | | | Streptococcus pneumoniae | 1 | Mycobacterium tuberculosis complex | 1 |
| | | | | Staphylococcus aureus | 1 | Mycobacterium mucogenicum | 1 |
| | | | | Staphylococcus hominis | 1 | Stenotrophomonas maltophilia | 1 |
| | | | | Stenotrophomonas maltophilia | 1 | Helicobacter pylori | 1 |
| | | | | Serratia marcescens | 1 | | |
| | | | | Campylobacter hominis | 1 | | |
| | | | | Clostridium tetani | 1 | | |
| | | | | Clostridium tertium | 1 | | |
| | | | | Mycobacterium tuberculosis complex | 1 | | |
| | | | | Mycobacterium mucogenicum | 1 | | |
| | | | | Mycoplasma hominis | 1 | | |
| | | | | Leptospiraspp. | 1 | | |
| | | | | Eubacterium limosum | 1 | | |
| | | | | Citrobacter sedlakii | 1 | | |

## Slide 2
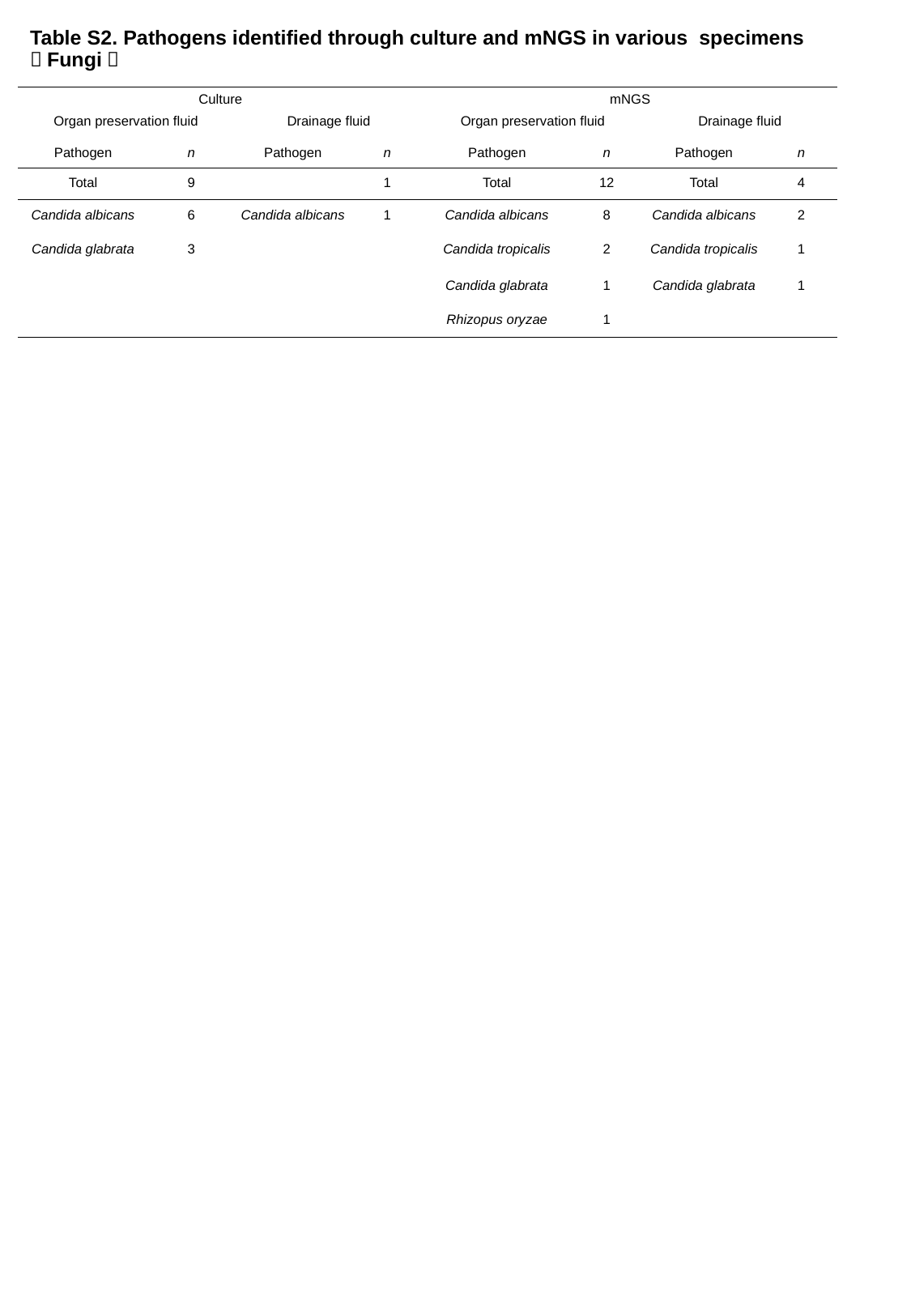

# Table S2. Pathogens identified through culture and mNGS in various specimens（Fungi）
| Culture | | | | mNGS | | | |
| --- | --- | --- | --- | --- | --- | --- | --- |
| Organ preservation fluid | | Drainage fluid | | Organ preservation fluid | | Drainage fluid | |
| Pathogen | n | Pathogen | n | Pathogen | n | Pathogen | n |
| Total | 9 | | 1 | Total | 12 | Total | 4 |
| Candida albicans | 6 | Candida albicans | 1 | Candida albicans | 8 | Candida albicans | 2 |
| Candida glabrata | 3 | | | Candida tropicalis | 2 | Candida tropicalis | 1 |
| | | | | Candida glabrata | 1 | Candida glabrata | 1 |
| | | | | Rhizopus oryzae | 1 | | |

## Slide 3
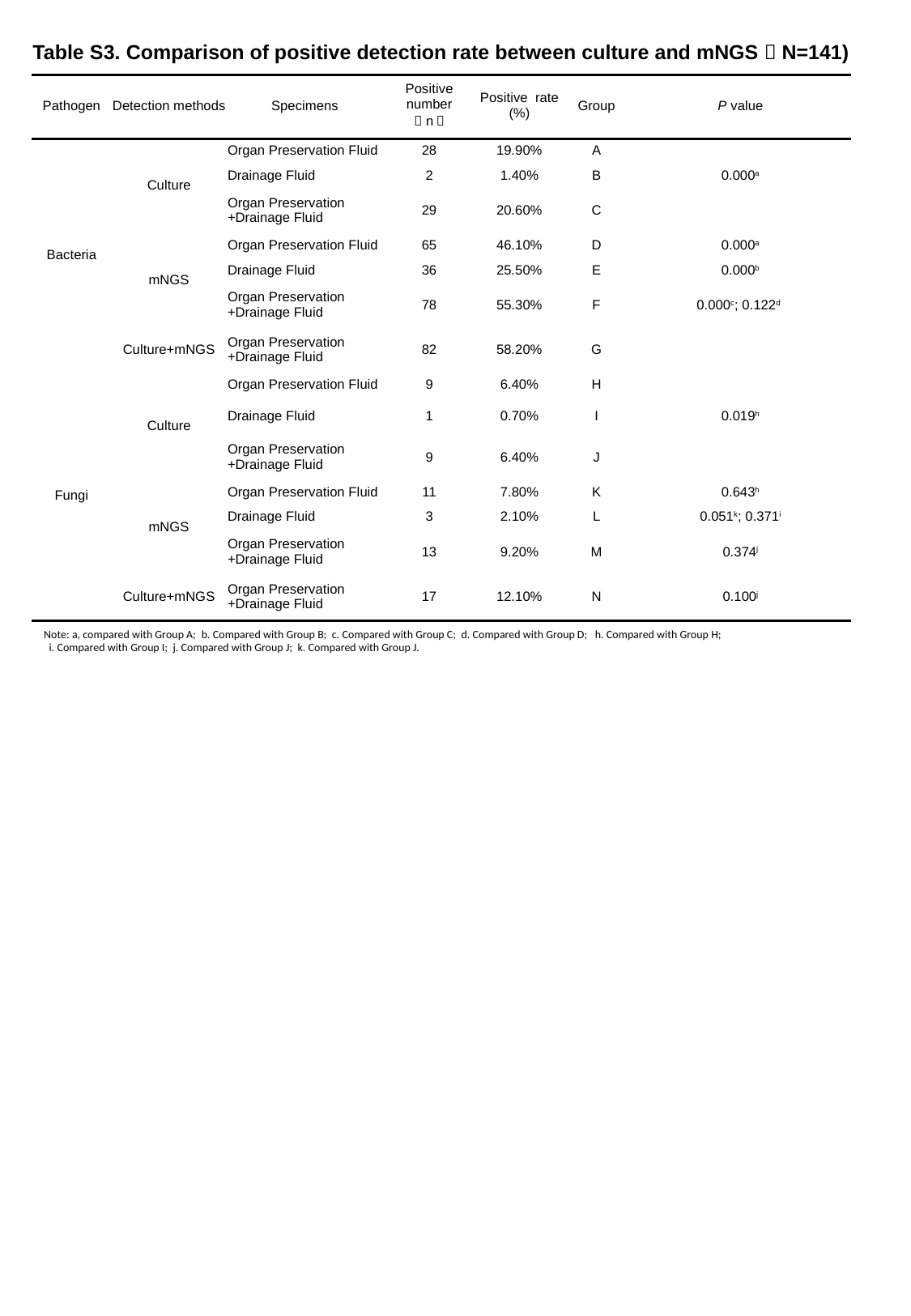

| Table S3. Comparison of positive detection rate between culture and mNGS（N=141) | | | | | | |
| --- | --- | --- | --- | --- | --- | --- |
| Pathogen | Detection methods | Specimens | Positive number （n） | Positive rate (%) | Group | P value |
| Bacteria | Culture | Organ Preservation Fluid | 28 | 19.90% | A | |
| | | Drainage Fluid | 2 | 1.40% | B | 0.000a |
| | | Organ Preservation +Drainage Fluid | 29 | 20.60% | C | |
| | mNGS | Organ Preservation Fluid | 65 | 46.10% | D | 0.000a |
| | | Drainage Fluid | 36 | 25.50% | E | 0.000b |
| | | Organ Preservation +Drainage Fluid | 78 | 55.30% | F | 0.000c; 0.122d |
| | Culture+mNGS | Organ Preservation +Drainage Fluid | 82 | 58.20% | G | |
| Fungi | Culture | Organ Preservation Fluid | 9 | 6.40% | H | |
| | | Drainage Fluid | 1 | 0.70% | I | 0.019h |
| | | Organ Preservation +Drainage Fluid | 9 | 6.40% | J | |
| | mNGS | Organ Preservation Fluid | 11 | 7.80% | K | 0.643h |
| | | Drainage Fluid | 3 | 2.10% | L | 0.051k; 0.371i |
| | | Organ Preservation +Drainage Fluid | 13 | 9.20% | M | 0.374j |
| | Culture+mNGS | Organ Preservation +Drainage Fluid | 17 | 12.10% | N | 0.100j |
Note: a, compared with Group A; b. Compared with Group B; c. Compared with Group C; d. Compared with Group D; h. Compared with Group H; i. Compared with Group I; j. Compared with Group J; k. Compared with Group J.

## Slide 4
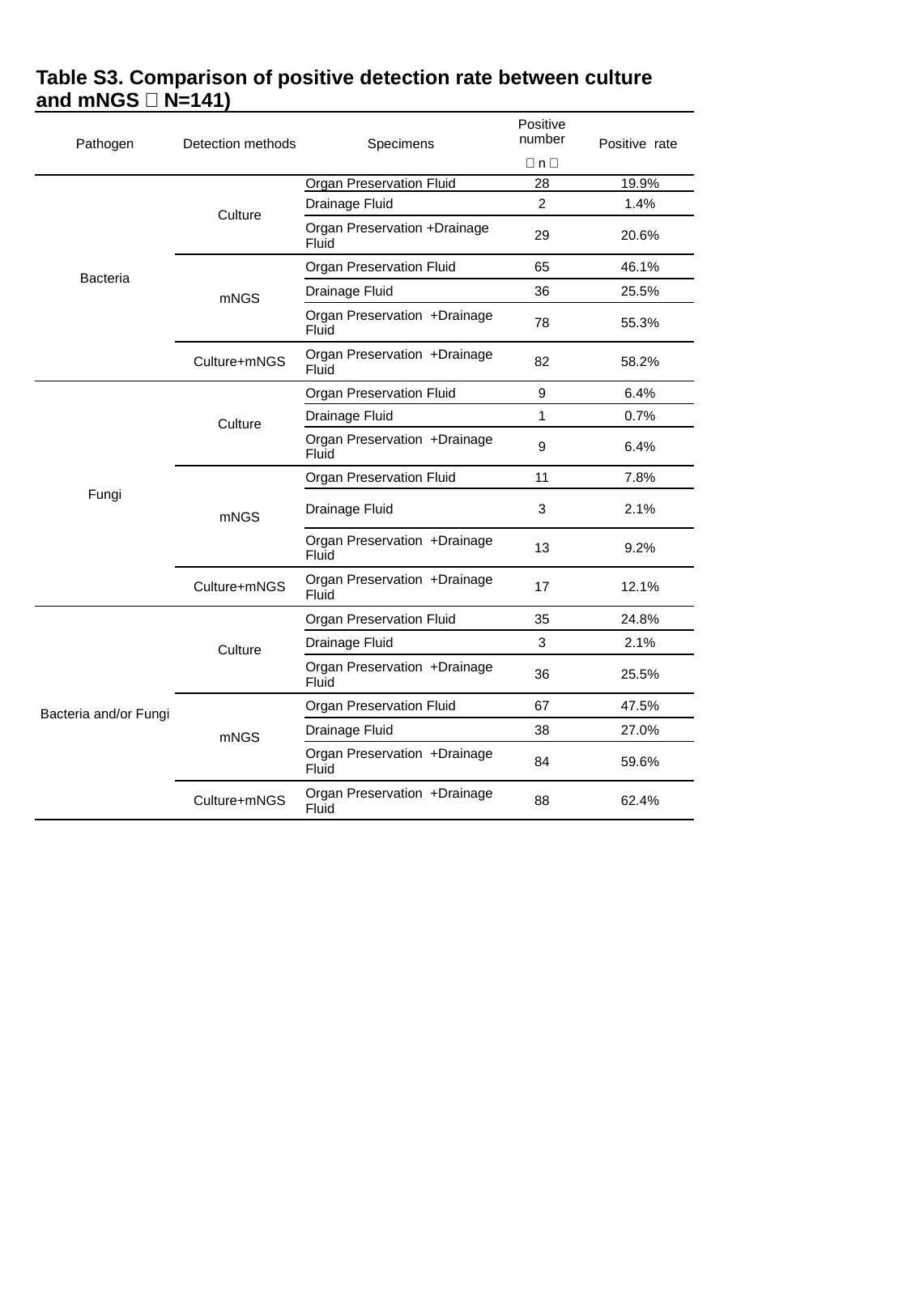

| Table S3. Comparison of positive detection rate between culture and mNGS（N=141) | | | | |
| --- | --- | --- | --- | --- |
| Pathogen | Detection methods | Specimens | Positive number | Positive rate |
| | | | （n） | |
| Bacteria | Culture | Organ Preservation Fluid | 28 | 19.9% |
| | | Drainage Fluid | 2 | 1.4% |
| | | Organ Preservation +Drainage Fluid | 29 | 20.6% |
| | mNGS | Organ Preservation Fluid | 65 | 46.1% |
| | | Drainage Fluid | 36 | 25.5% |
| | | Organ Preservation +Drainage Fluid | 78 | 55.3% |
| | Culture+mNGS | Organ Preservation +Drainage Fluid | 82 | 58.2% |
| Fungi | Culture | Organ Preservation Fluid | 9 | 6.4% |
| | | Drainage Fluid | 1 | 0.7% |
| | | Organ Preservation +Drainage Fluid | 9 | 6.4% |
| | mNGS | Organ Preservation Fluid | 11 | 7.8% |
| | | Drainage Fluid | 3 | 2.1% |
| | | Organ Preservation +Drainage Fluid | 13 | 9.2% |
| | Culture+mNGS | Organ Preservation +Drainage Fluid | 17 | 12.1% |
| Bacteria and/or Fungi | Culture | Organ Preservation Fluid | 35 | 24.8% |
| | | Drainage Fluid | 3 | 2.1% |
| | | Organ Preservation +Drainage Fluid | 36 | 25.5% |
| | mNGS | Organ Preservation Fluid | 67 | 47.5% |
| | | Drainage Fluid | 38 | 27.0% |
| | | Organ Preservation +Drainage Fluid | 84 | 59.6% |
| | Culture+mNGS | Organ Preservation +Drainage Fluid | 88 | 62.4% |

## Slide 5
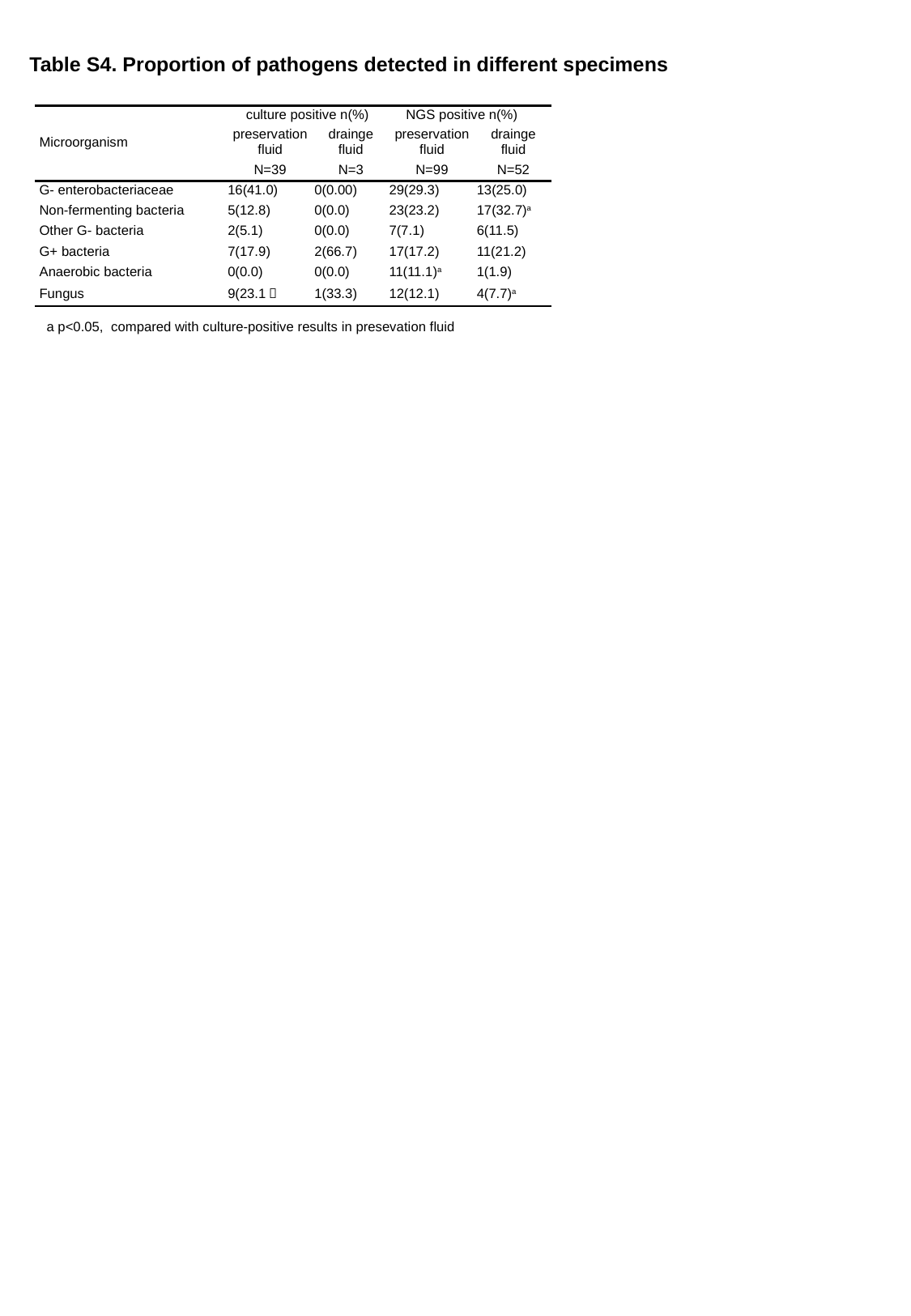

# Table S4. Proportion of pathogens detected in different specimens
| | | | | |
| --- | --- | --- | --- | --- |
| | culture positive n(%) | | NGS positive n(%) | |
| Microorganism | preservation fluid | drainge fluid | preservation fluid | drainge fluid |
| | N=39 | N=3 | N=99 | N=52 |
| G- enterobacteriaceae | 16(41.0) | 0(0.00) | 29(29.3) | 13(25.0) |
| Non-fermenting bacteria | 5(12.8) | 0(0.0) | 23(23.2) | 17(32.7)a |
| Other G- bacteria | 2(5.1) | 0(0.0) | 7(7.1) | 6(11.5) |
| G+ bacteria | 7(17.9) | 2(66.7) | 17(17.2) | 11(21.2) |
| Anaerobic bacteria | 0(0.0) | 0(0.0) | 11(11.1)a | 1(1.9) |
| Fungus | 9(23.1） | 1(33.3) | 12(12.1) | 4(7.7)a |
a p<0.05, compared with culture-positive results in presevation fluid

## Slide 6
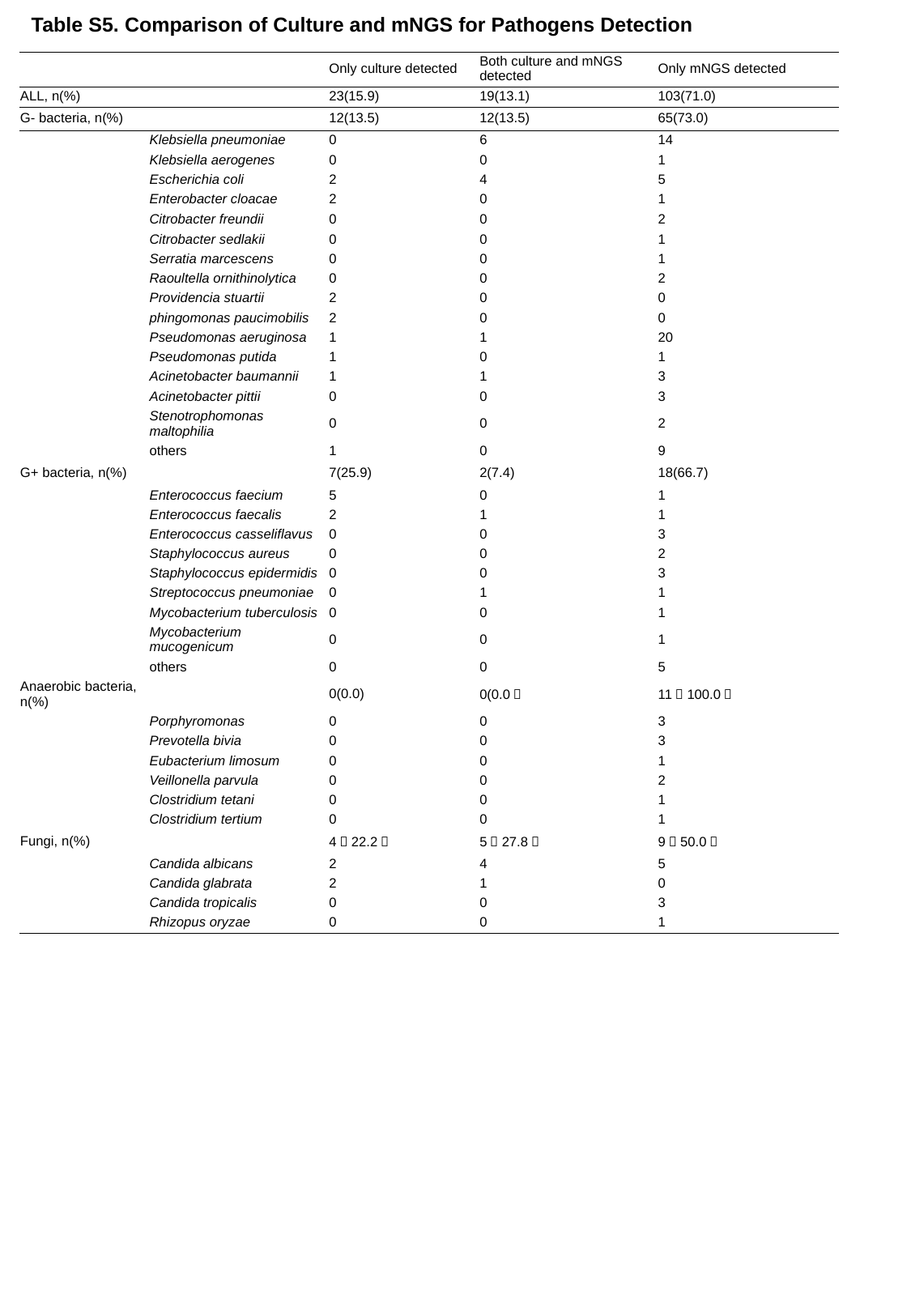

Table S5. Comparison of Culture and mNGS for Pathogens Detection
| | | Only culture detected | Both culture and mNGS detected | Only mNGS detected |
| --- | --- | --- | --- | --- |
| ALL, n(%) | | 23(15.9) | 19(13.1) | 103(71.0) |
| G- bacteria, n(%) | | 12(13.5) | 12(13.5) | 65(73.0) |
| | Klebsiella pneumoniae | 0 | 6 | 14 |
| | Klebsiella aerogenes | 0 | 0 | 1 |
| | Escherichia coli | 2 | 4 | 5 |
| | Enterobacter cloacae | 2 | 0 | 1 |
| | Citrobacter freundii | 0 | 0 | 2 |
| | Citrobacter sedlakii | 0 | 0 | 1 |
| | Serratia marcescens | 0 | 0 | 1 |
| | Raoultella ornithinolytica | 0 | 0 | 2 |
| | Providencia stuartii | 2 | 0 | 0 |
| | phingomonas paucimobilis | 2 | 0 | 0 |
| | Pseudomonas aeruginosa | 1 | 1 | 20 |
| | Pseudomonas putida | 1 | 0 | 1 |
| | Acinetobacter baumannii | 1 | 1 | 3 |
| | Acinetobacter pittii | 0 | 0 | 3 |
| | Stenotrophomonas maltophilia | 0 | 0 | 2 |
| | others | 1 | 0 | 9 |
| G+ bacteria, n(%) | | 7(25.9) | 2(7.4) | 18(66.7) |
| | Enterococcus faecium | 5 | 0 | 1 |
| | Enterococcus faecalis | 2 | 1 | 1 |
| | Enterococcus casseliflavus | 0 | 0 | 3 |
| | Staphylococcus aureus | 0 | 0 | 2 |
| | Staphylococcus epidermidis | 0 | 0 | 3 |
| | Streptococcus pneumoniae | 0 | 1 | 1 |
| | Mycobacterium tuberculosis | 0 | 0 | 1 |
| | Mycobacterium mucogenicum | 0 | 0 | 1 |
| | others | 0 | 0 | 5 |
| Anaerobic bacteria, n(%) | | 0(0.0) | 0(0.0） | 11（100.0） |
| | Porphyromonas | 0 | 0 | 3 |
| | Prevotella bivia | 0 | 0 | 3 |
| | Eubacterium limosum | 0 | 0 | 1 |
| | Veillonella parvula | 0 | 0 | 2 |
| | Clostridium tetani | 0 | 0 | 1 |
| | Clostridium tertium | 0 | 0 | 1 |
| Fungi, n(%) | | 4（22.2） | 5（27.8） | 9（50.0） |
| | Candida albicans | 2 | 4 | 5 |
| | Candida glabrata | 2 | 1 | 0 |
| | Candida tropicalis | 0 | 0 | 3 |
| | Rhizopus oryzae | 0 | 0 | 1 |

## Slide 7
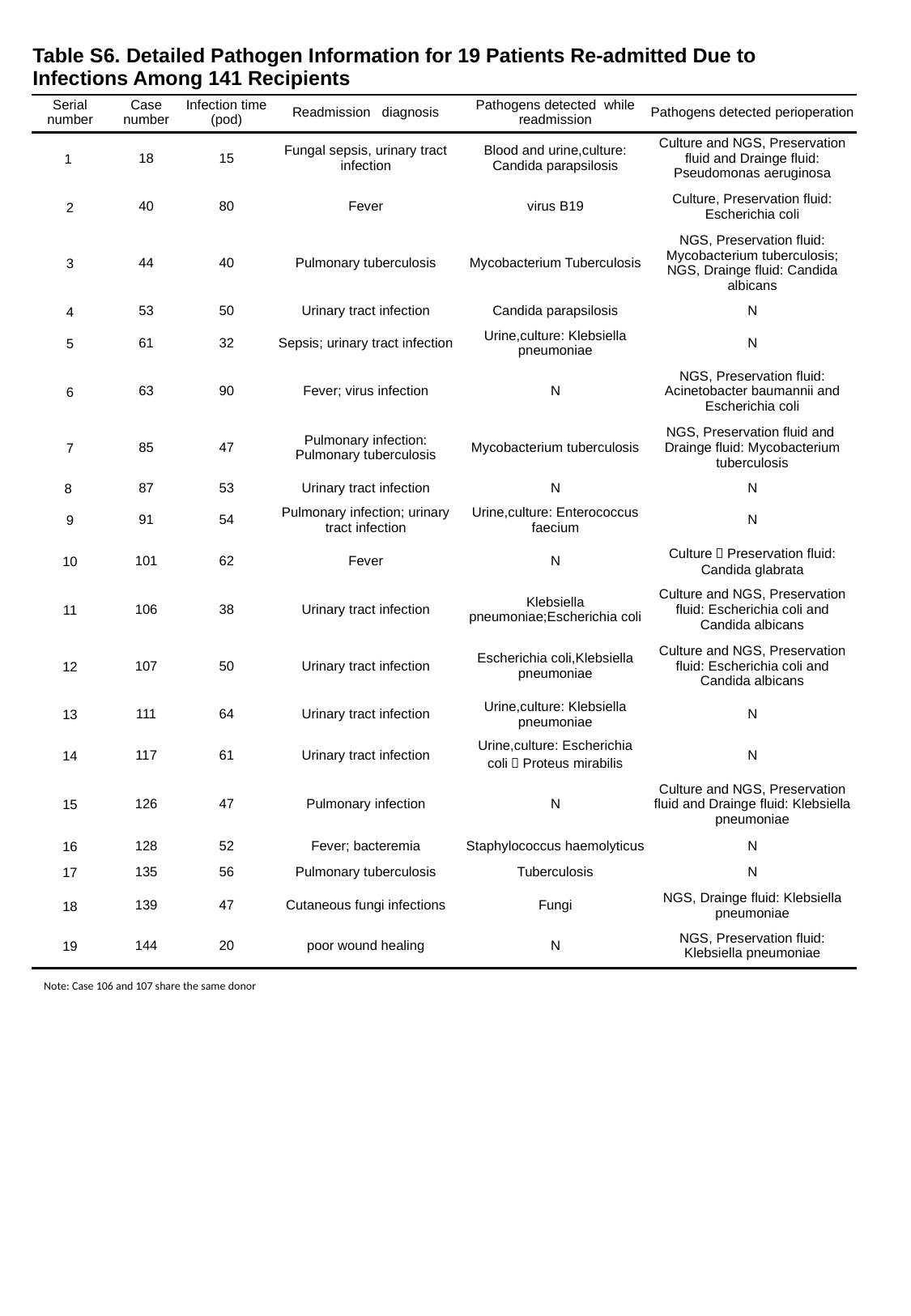

| Table S6. Detailed Pathogen Information for 19 Patients Re-admitted Due to Infections Among 141 Recipients | | | | | |
| --- | --- | --- | --- | --- | --- |
| Serial number | Case number | Infection time (pod) | Readmission diagnosis | Pathogens detected while readmission | Pathogens detected perioperation |
| 1 | 18 | 15 | Fungal sepsis, urinary tract infection | Blood and urine,culture: Candida parapsilosis | Culture and NGS, Preservation fluid and Drainge fluid: Pseudomonas aeruginosa |
| 2 | 40 | 80 | Fever | virus B19 | Culture, Preservation fluid: Escherichia coli |
| 3 | 44 | 40 | Pulmonary tuberculosis | Mycobacterium Tuberculosis | NGS, Preservation fluid: Mycobacterium tuberculosis; NGS, Drainge fluid: Candida albicans |
| 4 | 53 | 50 | Urinary tract infection | Candida parapsilosis | N |
| 5 | 61 | 32 | Sepsis; urinary tract infection | Urine,culture: Klebsiella pneumoniae | N |
| 6 | 63 | 90 | Fever; virus infection | N | NGS, Preservation fluid: Acinetobacter baumannii and Escherichia coli |
| 7 | 85 | 47 | Pulmonary infection: Pulmonary tuberculosis | Mycobacterium tuberculosis | NGS, Preservation fluid and Drainge fluid: Mycobacterium tuberculosis |
| 8 | 87 | 53 | Urinary tract infection | N | N |
| 9 | 91 | 54 | Pulmonary infection; urinary tract infection | Urine,culture: Enterococcus faecium | N |
| 10 | 101 | 62 | Fever | N | Culture，Preservation fluid: Candida glabrata |
| 11 | 106 | 38 | Urinary tract infection | Klebsiella pneumoniae;Escherichia coli | Culture and NGS, Preservation fluid: Escherichia coli and Candida albicans |
| 12 | 107 | 50 | Urinary tract infection | Escherichia coli,Klebsiella pneumoniae | Culture and NGS, Preservation fluid: Escherichia coli and Candida albicans |
| 13 | 111 | 64 | Urinary tract infection | Urine,culture: Klebsiella pneumoniae | N |
| 14 | 117 | 61 | Urinary tract infection | Urine,culture: Escherichia coli，Proteus mirabilis | N |
| 15 | 126 | 47 | Pulmonary infection | N | Culture and NGS, Preservation fluid and Drainge fluid: Klebsiella pneumoniae |
| 16 | 128 | 52 | Fever; bacteremia | Staphylococcus haemolyticus | N |
| 17 | 135 | 56 | Pulmonary tuberculosis | Tuberculosis | N |
| 18 | 139 | 47 | Cutaneous fungi infections | Fungi | NGS, Drainge fluid: Klebsiella pneumoniae |
| 19 | 144 | 20 | poor wound healing | N | NGS, Preservation fluid: Klebsiella pneumoniae |
Note: Case 106 and 107 share the same donor
